# State-of-the-art advances in tissue-engineered corneal substitutes: a systematic review (2020–2025)

**DOI:** 10.1177/25158414261464536

**Published:** 2026-08-06

**Authors:** Mario Bonmatí-Echevarría, Carmen González-Gallardo, Miguel Alaminos

**Affiliations:** Division of Ophthalmology, University Hospital Clínico San Cecilio, Granada, Spain; Division of Ophthalmology, University Hospital Clínico San Cecilio, Granada, Spain; Department of Histology, Tissue Engineering Group, School of Medicine, University of Granada, Granada, Spain; Instituto de Investigación Biosanitaria ibs.GRANADA, Granada, Spain; Department of Histology, Tissue Engineering Group, School of Medicine, University of Granada, Granada, Spain; Instituto de Investigación Biosanitaria ibs.GRANADA, Granada, Spain

**Keywords:** artificial cornea, bioengineering, corneal substitutes, corneal tissue engineering, keratoprosthesis, regenerative ophthalmology

## Abstract

**Background:**

Corneal blindness remains a major global health burden, limited by donor shortage and graft-related complications. Tissue-engineered corneal substitutes have emerged as a promising alternative, aiming to restore corneal structure and function through bioengineered constructs.

**Objectives:**

To systematically review recent advances (2020–2025) in tissue-engineered corneal substitutes, classifying them according to the corneal layer replaced and evaluating their biological, optical, and functional performance, as well as their translational potential for clinical application.

**Design:**

A systematic review.

**Data sources and methods:**

A systematic review was conducted following Preferred Reporting Items for Systematic Review and Meta-Analyses (PRISMA) 2020 guidelines. Searches were performed in PubMed/MEDLINE, Scopus, and Web of Science using predefined keywords related to corneal tissue engineering. Eligible studies included experimental, preclinical, or clinical studies published between January 2020 and June 2025 describing cell-based corneal substitutes. Studies focused on keratoprostheses (KPros), acellular scaffolds, or non-cellular biomaterials were excluded.

**Results:**

Fourteen studies met the inclusion criteria: five endothelial, three stromal, one epithelial, and five epithelium–stroma substitutes. Endothelial models demonstrated cell viability, expression of tight junction proteins (ZO-1, Na^+^/K^+^-ATPase), and partial restoration of corneal transparency in animal and ex vivo systems. Stromal models incorporated advanced biofabrication techniques such as 3D bioprinting and neuronal co-culture, achieving > 80% optical transmittance and adequate biomechanical properties. The single epithelial model achieved complete re-epithelialization in a rabbit limbal deficiency model. Multilayered epithelium–stroma substitutes, including the NANOULCOR (Tissue Engineering Group, University of Granada, Granada, Spain) construct, exhibited safety and feasibility in preclinical and early clinical studies.

**Conclusion:**

Recent progress in corneal tissue engineering has yielded increasingly functional and biocompatible substitutes that replicate native corneal architecture. However, most studies remain limited by small sample sizes, short follow-up, and reliance on animal models. Further standardized clinical trials are required for clinical translation.

**Trial registration:**

Not applicable.

## Plain language summary

New laboratory-made corneal tissues that could replace damaged corneas in the future: a review of recent scientific advances

Corneal diseases are one of the main causes of vision loss worldwide. Many patients can recover their sight through a corneal transplant, but donated corneas are limited and not always suitable. For this reason, scientists are developing new laboratory-made corneal tissues using advanced techniques in tissue engineering. These artificial corneal substitutes could one day help patients who cannot receive a standard transplant. This article reviews the most important scientific advances made between 2020 and 2025 in the creation of tissue-engineered corneal substitutes. The studies included in this review explored different ways of rebuilding the cornea depending on the layer that is damaged: the epithelium (the surface), the stroma (the middle layer), or the endothelium (the inner layer). Some research groups also created substitutes that combine several layers at once. The methods used to create these tissues include 3D bioprinting, modified biological scaffolds, stem cells, and ultra-thin natural membranes. In laboratory tests and animal studies, many of these substitutes showed good transparency, healthy cell growth, and the ability to integrate with the host cornea. One model, called NANOULCOR, has already been tested in a small group of patients with severe corneal disease and showed promising safety and healing results. Although the progress is encouraging, most studies were small and had short follow-up periods. More research is needed to confirm long-term safety, transparency, and vision outcomes before these substitutes can become a routine treatment for patients. This review provides an accessible overview of how tissue-engineered corneal substitutes are evolving and how they may help address the global shortage of donor corneas in the coming years.

## Introduction

The cornea is one of the most densely innervated and transparent tissues of the human body. It is avascular and located in the anterior segment of the eye, playing a fundamental role both as a structural and protective component and by contributing nearly two-thirds of the eye’s total refractive power.^1,2^ Histologically, the cornea consists of three cellular layers—the epithelium, stroma, and endothelium—and two acellular layers, Bowman’s and Descemet’s membranes.^2^ The outermost layer, the epithelium, represents approximately 10% of the corneal thickness and is composed of stratified epithelial cells firmly attached to a basal membrane, providing the first barrier of protection. Beneath it lies Bowman’s membrane, an acellular layer that supports epithelial adhesion and transitions into the stroma. The stroma constitutes 80%–85% of corneal thickness and is composed of keratocytes embedded within a dense extracellular matrix rich in collagen and glycosaminoglycans.^1,2^ Descemet’s membrane, primarily composed of type IV and VIII collagen and laminin, underlies the stroma and serves as the basement membrane for the endothelium—a monolayer of metabolically active cells that regulate corneal hydration through ionic pumps but lack regenerative capacity in adults.¹

In addition to these five layers, some authors have described a sixth, the so-called Dua’s layer, located between the stroma and Descemet’s membrane.^3^ This acellular layer is believed to contribute to corneal biomechanics and has surgical relevance in posterior lamellar keratoplasty techniques.

Corneal diseases are the fifth leading cause of blindness worldwide, accounting for approximately 12% of all global cases and affecting around six million people.^4–6^ In Western countries, hereditary, degenerative, and iatrogenic disorders—such as keratoconus and Fuchs endothelial dystrophy—predominate, whereas in developing regions (mainly in Asia and Africa), infectious and nutritional corneal diseases are more prevalent.^4^

Due to its avascular structure and limited regenerative capacity, corneal integrity is difficult to restore after injury or disease. When severe opacities compromise visual acuity, corneal transplantation (keratoplasty) remains the treatment of choice.^2,4^ Primary corneal grafts—either full-thickness or lamellar—show survival rates of 87%–93% at 1 year and 72%–73% at 5 years in non-complex eyes, such as those with keratoconus or bullous keratopathy.^2,5^ However, graft survival decreases over time, especially in cases with recurrent inflammation, glaucoma, infection, or neovascularization, often requiring repeat transplantation.²

Modern keratoplasty includes both full-thickness (penetrating) and lamellar techniques, such as deep anterior lamellar keratoplasty (DALK) and posterior procedures like Descemet stripping automated endothelial keratoplasty and Descemet membrane endothelial keratoplasty.^4^ Despite surgical advances, the global shortage of donor corneas remains a critical limitation, prompting the need for innovative alternatives.

One promising approach involves the development of tissue-engineered corneal substitutes through tissue engineering, producing biological substitutes that restore, maintain, or improve corneal function.^2^ A tissue-engineered corneal substitute is a laboratory-generated substitute designed to replicate the properties of the native human cornea, composed of three basic elements: (1) human cells, (2) biocompatible biomaterials, and (3) growth and differentiation factors.^7^

As a separate therapeutic approach, KPros—synthetic optical devices made of polymethylmethacrylate with a structural skirt of various materials such as titanium, Teflon, osteodental lamina, or bone—serve as a last-resort option for end-stage corneal blindness.² The Boston type I KPro (Massachusetts Eye and Ear Infirmary, Boston, MA, USA) remains the most widely used artificial corneal implant worldwide.^5^ Although these devices reduce immunologic rejection and provide good optical properties, they require complex surgery and carry risks of postoperative complications such as extrusion, infection, and particularly glaucoma, which is the leading cause of vision loss after KPro implantation.^2,8^ Infectious complications such as keratitis and endophthalmitis have also been reported, with prevalence rates up to 17.3% and 12.5%, respectively.^9^

Given these limitations, tissue-engineered corneal substitutes have emerged as a safer and more biocompatible alternative, minimizing the risks of infection and glaucoma associated with KPros. Although clinical use remains limited, preliminary studies have demonstrated the safety and feasibility of these bioengineered corneal constructs in humans.^10^ These tissue-engineered models aim to reproduce the structure and physiology of the native cornea using biological scaffolds such as human amniotic membrane (AM), decellularized tissues, or hydrogels.^11^ Several of these constructs have shown regenerative and optical properties comparable to the human cornea, opening new therapeutic avenues for corneal diseases.^11^

## Materials and methods

### Search strategy

A systematic review of the scientific literature was conducted and reported in accordance with PRISMA guidelines.^12^ The completed PRISMA checklist is provided as Supplemental Material.

The databases PubMed/MEDLINE, Scopus, and Web of Science (WoS) were searched in June 2025. The search strategy combined the following terms and Boolean operators:

“cornea” AND “tissue engineering”, “artificial cornea” AND “scaffold”, and “tissue scaffolds” AND “cornea”.

Equivalent search strategies were adapted as necessary for each database while maintaining the same search concepts and Boolean operators. Only studies published between January 2020 and June 2025 were considered. The reference lists of relevant articles were also screened manually to identify additional eligible studies.

#### Inclusion and exclusion criteria

Eligibility criteria were defined a priori according to the objectives of this systematic review and applied consistently during the study selection process.

Studies were included if they met all the following criteria:

- Study type: Original experimental, preclinical (*in vitro* or *in vivo*), or clinical studies.
- Intervention: Development of tissue-engineered corneal substitutes defined as bioengineered constructs combining living cells with a biomaterial scaffold.
- Biological relevance: Constructs intended to replace, regenerate, or functionally mimic at least one corneal layer (epithelium, stroma, and/or endothelium).
- Cellular component: Use of viable cells (e.g., epithelial cells, keratocytes, endothelial cells, stem cells, or induced pluripotent stem cell–derived cells) integrated within the scaffold.
- Publication criteria: Articles published in peer-reviewed journals between January 2020 and June 2025.
- Language: Publications in English or Spanish.

Studies were excluded if they met any of the following criteria:

- Acellular constructs: Studies describing biomaterials or scaffolds without incorporation of living cells.
- Keratoprostheses (KPros): Studies focused on synthetic, non-biological artificial corneas or prosthetic devices without a tissue-engineered cellular component.
- Non-structural approaches: Studies addressing corneal epithelial wound healing or surface repair without the development of a structured corneal substitute intended to replicate one or more corneal layers.
- Non-original studies: Reviews, systematic reviews, meta-analyses, editorials, letters, conference abstracts, or case reports without original experimental data.
- Irrelevant scope: Studies not primarily focused on corneal tissue engineering (e.g., general biomaterials research without corneal application).

Publication date and language: Articles published before 2020 or in languages other than English or Spanish. These criteria were designed to ensure the inclusion of studies specifically addressing biologically relevant, cell-based corneal substitutes while excluding approaches lacking translational potential within the scope of regenerative ophthalmology.

### Study selection

The study selection process was conducted in accordance with PRISMA 2020 guidelines.

All records identified through database searching were exported, and duplicates were removed. The remaining studies underwent a two-stage screening process.

In the first stage, titles and abstracts were independently screened by two reviewers to assess potential eligibility based on the predefined inclusion and exclusion criteria. Studies that clearly did not meet the eligibility criteria were excluded at this stage.

In the second stage, full-text articles of the remaining studies were retrieved and assessed independently by the same two reviewers. Reasons for exclusion at the full-text stage were documented and included, among others: absence of a cellular component, lack of a tissue-engineered corneal construct, focus on keratoprostheses, or insufficient relevance to corneal tissue engineering.

Any discrepancies between reviewers during both screening phases were resolved through discussion and consensus. When necessary, a third reviewer was consulted to reach a final decision.

**Figure 1.**
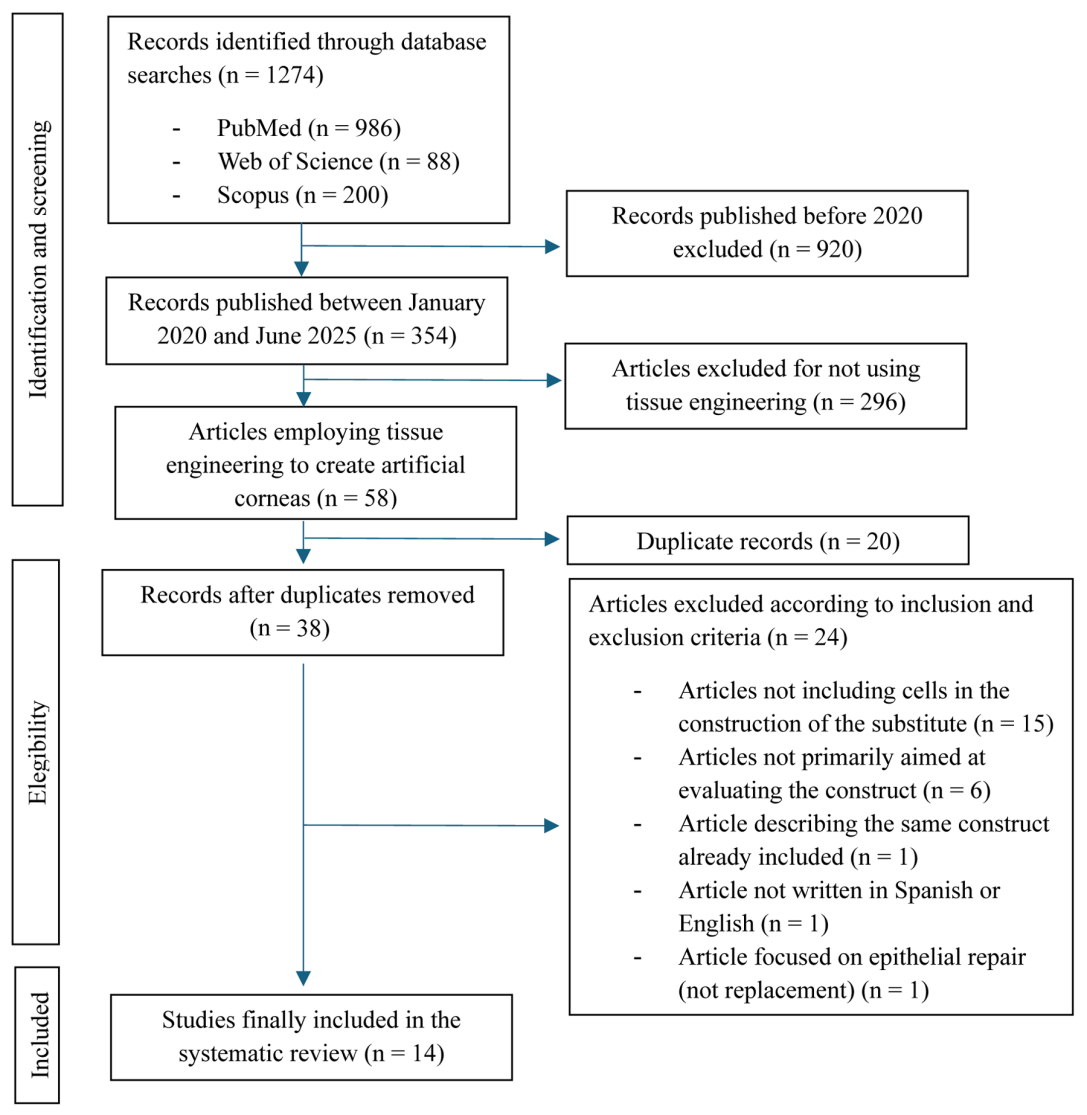
Flowchart illustrating the study selection process for the present systematic review.

The overall selection process, including identification, screening, eligibility, and inclusion, is summarized in a PRISMA 2020 flow diagram in Figure 1. Finally, 14 studies were selected for qualitative analysis.

### Sample size considerations

No formal sample size calculation or power analysis was performed, as this study is a systematic review. The number of included studies was determined by the predefined search strategy, eligibility criteria, and study selection process in accordance with PRISMA 2020 guidelines. Therefore, the final sample size reflects the total number of studies meeting the inclusion criteria within the specified time frame.

### Data extraction and classification

Data were extracted regarding:

- Type of corneal substitute developed (endothelial, stromal, epithelial, or epithelium–stroma).
- Biomaterials employed and their physicochemical properties.
- Cell sources and differentiation strategies.
- Functional, optical, and biomechanical outcomes (in vitro, ex vivo, and in vivo).
- Limitations and translational perspectives reported by each study.

The studies were subsequently grouped into four categories according to the corneal layer replaced to facilitate comparative analysis:

1. Endothelial substitutes.
2. Stromal substitutes.
3. Epithelial substitutes.
4. Epithelium–stroma composite constructs.

Due to the high heterogeneity of the included studies in terms of experimental design, biomaterials, cell sources, and outcome assessment methods, a formal risk of bias assessment, reporting bias evaluation, and certainty of evidence grading (e.g., GRADE) were not performed. The review was therefore conducted as a qualitative synthesis, focusing on descriptive and comparative analysis of the available evidence.

## Results

Tissue-engineered corneal constructs were first classified according to the native corneal layer they are intended to replace. The comparative summary of their optical and biological performance is shown in Tables 1 to 3.

**Table 1.** Comparative summary of endothelial corneal substitutes.

Study	Evidence level and model type	Follow-up	Composition of corneal substitute	Main results	Main limitations
Tsai (2025)	Preclinical. Ex vivo (human cornea)	14 days	Type I collagen hydrogel (RAFT) + porcine endothelial cells	Functional grafts; good transparency; adequate morphology and viability	Short follow-up; small sample (*n* = 3); porcine cells; no in vivo validation
Zhang (2022)	Preclinical. In vivo (rabbits)	Up to 28 days	Ultrathin acellular porcine corneal stroma + hCECs	Good adhesion and transparency; corneal thickness restored	Number of animals not specified; no long-term data
An (2020)	Preclinical. In vitro	7 days	Decellularized porcine Descemet membranes + hPSC-derived hCECs	Stable in vitro cellularized graft; adequate phenotypic expression	No in vivo validation; long-term transparency not assessed
Lu (2020)	Preclinical. In vivo (cat)	98 days	Porcine Descemet membranes + human CECs	Successful implantation; maintained transparency and corneal thickness	Number of animals not specified; no functional marker analysis; no immunogenicity data
Tayebi (2021)	Preclinical. In vitro	10 days	Chitosan/chitosan-nanoparticle/polycaprolactone (CSNP/PCL 50/25) membrane	High transparency (~74%); good hCEC adhesion and phenotype preserved (CD166^+^, CD44^–^)	No in vivo data, short follow-up; no functional or surgical mechanical testing

**Table 2.** Comparative summary of stromal corneal substitutes.

Study	Evidence level and model type	Follow-up	Composition of corneal substitute	Main results	Main limitations
Mörö (2022)	Preclinical. In vitro + ex vivo (porcine)	21 days	Bio-ink of hyaluronic acid (HA) + GelMA and collagen fibers + hCSSCs and neuronal hPSCs	Cell viability > 90%; keratocyte markers maintained; neuronal co-culture enabled axonal growth into stroma; high optical transparency (>80%)	No in vivo data; no immunological evaluation; mechanical resistance not tested
Fernández-Pérez (2021)	Preclinical. In vivo (rabbit)	3 months	Decellularized porcine corneas + primary human keratocytes	Structural integrity and partial transparency; lower inflammation and opacity in recellularized vs. acellular grafts; organized host cell infiltration; reduced neovascularization	Animal model; small sample; short follow-up; persistent neovascularization in some cases; no innervation evaluation
Vijayaraghavan (2024)	Preclinical. In vitro	14 days	3D-bioprinted GelMA/agarose (0.5–1% w/w) hydrogel + goat stromal keratocytes	80% transparency; high viability and proliferation; expression of keratan sulfate and vimentin; better cohesion with higher agarose content	No in vivo data; goat cells used; transparency and mechanics evaluated only under static conditions; no long-term degradation study

**Table 3.** Comparative summary of epithelium–stroma corneal.

Study	Evidence level and model type	Follow-up	Composition of corneal substitute	Main results	Main limitations
Ortiz-Arrabal (2024) González Gallardo (2023)	Clinical Trial (Phase I-IIA). Human.	24 months	NANOULCOR: nanostructured fibrin-agarose biomaterial + corneal epithelial cells and keratocytes	Stratified epithelium with adequate differentiation and integrity; organized, homogeneous stroma; good transparency (~80%); no ulcer recurrence or rescue surgery; no rejection, neovascularization, infection, or graft detachment; safe surgical handling and suture resistance	Low stromal maturation; small in vivo sample (five patients)
Bhutani (2022)	Preclinical. In vitro.	21 days	DLP-bioprinted biopolymeric corneal lenticules composed of GelMA and methacrylated collagen + human stromal cells	Cell viability > 90%; mechanical properties comparable to native cornea; transparency > 85%; good proliferation and elongation on scaffold; adequate water retention	In vitro only
Xie (2020)	Preclinical. In vivo	12 months	Bioengineered stromal implants from allogeneic cornea-derived matrix + hCECs	Good biomechanical properties and biocompatibility; maintained transparency; positive expression of collagen and keratocyte markers	Animal model physiologically different from humans; small sample size
Garzón (2020)	Preclinical. In vivo (rabbit)	12 months	Agarose hydrogels + hCECs, hCSCs/HWJSCs	Both substitutes developed well-stratified epithelium (~6.5 layers) and dense, organized stroma; no rejection or infection; increasing transparency over time, higher in orthotypic model	Animal model; small sample; no direct comparison with approved substitutes

### Endothelial corneal substitutes

In recent years, multiple tissue-engineering approaches have been developed to reconstruct a functional corneal endothelium as an alternative to corneal transplantation. In this review, we identified five articles in which authors engineered endothelial substitutes by combining different biomaterials with diverse components and cell types, assessing structural and functional viability in vitro and/or in vivo,

Tsai et al. developed an ex vivo human cornea model using a compressed type I collagen matrix (RAFT, Real Architecture For 3D Tissues, TAP Biosystems, Royston, UK) seeded with porcine corneal endothelial cells to evaluate viability and function.^13^

Zhang et al. generated endothelial sheets on ultrathin acellular porcine stromal substrates, seeding rabbit corneal endothelial cells (rCECs) and human corneal endothelial cells (hCECs) for endothelial keratoplasty, and confirmed integration and functionality in vitro and in vivo.^14^

Tayebi et al. fabricated transparent chitosan/chitosan-nanoparticle/polycaprolactone (PCL) membranes for corneal endothelial engineering, seeded them with hCECs, and analyzed physical properties and cell compatibility.^15^

An et al. created ultrathin Descemet layers by decellularizing porcine corneas and seeding them with induced pluripotent stem cell-derived corneal endothelial-like cells (iPSC-hCECs) for ex vivo testing.^16^

Lu et al. reconstructed human corneal endothelial sheets on porcine Descemet membrane and implanted them in cats to evaluate the recovery of corneal transparency and cell density in vivo.^17^

#### Types of biomaterials used

All studies used biological materials as scaffolds. Tsai et al. developed a compressed type I collagen RAFT matrix that proved translucent and mechanically stable.^13^ Zhang et al. used acellular porcine stroma cut into 20-µm ultrathin lamellae, optimizing cell viability and facilitating surgical implantation.^14^ Tayebi et al. engineered scaffolds using different chitosan/PCL ratios.^15^ An et al. produced an ultrathin Descemet membrane graft derived from porcine cornea; decellularization preserved >90% of native tissue transparency.^16^ Finally, Lu et al. used porcine Descemet membranes treated with ethylene glycol diglycidyl ether, maintaining physical properties and enhancing cell adhesion.^17^

#### Cell types and sources

All five studies used different cell sources to cellularize the scaffolds. Tsai et al. used porcine corneal endothelial cells (pCECs).^13^ Zhang et al. seeded both rCECs and hCECs to validate results.^14^ Tayebi et al. employed hCECs.^15^ An et al. introduced an iPSC-based strategy, differentiating cells toward corneal endothelial phenotype—potentially autologous and clinically relevant.^16^ Lu et al. used a human-origin cell line that showed chromosomal stability and absence of tumorigenic transformation.^17^

#### Cell viability and organization

Across studies, seeded cells formed organized endothelial monolayers with hexagonal/polygonal morphology and expressed characteristic markers such as ZO-1 and Na⁺/K⁺-ATPase. No migration or detachment was observed in ex vivo or animal models.

All reported acceptable cell densities on scaffolds: Tsai et al. ~3000 cells/mm² with good viability after 14 days ex vivo^13^; Zhang et al. 3726 ± 223 cells/mm^2^ at day 5^14^; Tayebi et al. documented appropriate hCEC–biomaterial interaction after 5 days^15^; An et al. confirmed survival and homogeneous distribution of iPSC-derived cells^16^; Lu et al. reported an initial density of 3020 cells/mm² maintained at 2521 cells/mm^2^ at 98 days post-implantation in a feline model.^17^

#### Functional assessment and optical properties

Functionally, most constructs showed notable ability to restore endothelial function in the evaluated models.

Tsai et al. observed a significant reduction in corneal thickness in the treated group (641 ± 57 µm) versus acellular RAFT controls (752 ± 49 µm), indicating restored endothelial pump activity.^13^ Tayebi et al. found that adding chitosan nanoparticles improved transparency, cell–material interaction, and membrane surface properties.^15^ Zhang et al. implanted the grafts in rabbits via endothelial keratoplasty; progressive transparency restoration was seen at day 7, with normalization of corneal thickness by day 28, confirming posterior stromal adhesion and graft functionality.^14^ An et al. assessed in vitro outcomes only, yet demonstrated viability, marker expression, and appropriate morphology after 7 days.^16^ Lu et al. transplanted the construct into cats, observing complete recovery of corneal transparency and thickness reduction to 525 ± 56 µm at day 98.^17^

#### Biocompatibility and immunogenicity

Studies using porcine material ensured removal of porcine antigens through published decellularization processes, with no evidence of immune infiltration or rejection. Grafts were well tolerated in culture and animal models, with no relevant complications reported.

#### Limitations

Despite meaningful advances, all five studies share limitations affecting interpretation and clinical extrapolation. Short follow-up is common: Tsai’s ex vivo model lasted 14 days; in vivo follow-up in Zhang and Lu was limited to ~14 and 98 days, respectively, precluding long-term stability assessment.^13,14,17^ An’s study was in vitro only (7 days after iPSC seeding), and Tayebi’s follow-up was limited to in vitro assays (7 days for proliferation; 10 days for flow-cytometry phenotype).^15,16^ Small sample sizes further restrict generalizability (e.g., Tsai used only three pairs of human corneas ex vivo).^13^ Animal sample sizes were not specified in Zhang and Lu, although functional follow-up was complete.^14,17^ Use of non-human cells (pCECs, rCECs) introduces physiological differences versus human cells; hCEC validation, when included, was limited or in vitro.^13,14^ Additionally, while the biomaterials (compressed collagen, porcine Descemet, acellular stroma) appear biocompatible and optically suitable, there are no clinical data on long-term degradation, human tissue interactions, or potential immune responses under physiological conditions. Quantitative transparency was explicitly reported in only three studies (Tsai, An, Tayebi); others provided qualitative assessments without analyzing light scatter, temporal loss of clarity, or direct comparison with native human cornea. Finally, most studies did not evaluate critical aspects such as secondary angiogenesis or interaction with aqueous humor.

### Stromal corneal substitutes

We analyzed three recent experimental studies focused on stromal regeneration using cellular constructs—biofabricated substitutes containing living cells. Each applied distinct tissue-engineering strategies:

Mörö et al. used 3D bioprinting to develop a human stromal model with innervation, based on a bio-ink of modified hyaluronic acid, gelatin methacrylate (GelMA), and type I collagen optimized for adipose-derived mesenchymal stem cells (hASCs) differentiated into keratocytes, together with sensory neurons derived from induced pluripotent stem cells, aiming to recreate a stromal microenvironment with neural integration.^18^

Fernández-Pérez et al. assessed the impact of prolonged pre-implant recellularization with keratocytes on decellularized porcine corneas in a rabbit anterior lamellar keratoplasty model, testing whether longer pre-culture improved integration, transparency, and remodeling.^19^

Vijayaraghavan et al. formulated a GelMA/agarose hydrogel suitable for 3D bioprinting, incorporating goat-derived stromal keratocytes, and analyzed physicochemical, optical, and cytocompatibility properties in vitro to mimic native keratocyte phenotype.^20^

Across the three studies, construct evaluation included morphological, biological, optical, mechanical, and—in some cases—in vivo functional parameters, enabling cross-comparison of strategies.

#### Types of biomaterials used

Mörö et al. created a 3D-bioprinted bio-ink matrix (modified HA, GelMA, type I collagen) with well-defined lamellar alignment and pre-formed channels to guide axonal growth.^18^ Fernández-Pérez et al. used decellularized porcine corneas preserving collagen fibrillar organization.^19^ Vijayaraghavan et al. generated 3D-printed GelMA/agarose stromal sheets; the printed structures showed interconnected porosity with mean pore diameters of ~75 µm (0.5% agarose) and ~55 µm (1% agarose), with higher agarose increasing stiffness and reducing pore size.^20^

#### Cell types and sources

Mörö et al. used hASCs differentiated into keratocytes plus iPSC-derived sensory neurons to create an innervated stromal substitute.^18^ Fernández-Pérez et al. recellularized decellularized porcine corneas with human keratocytes.^19^ Vijayaraghavan et al. incorporated goat-derived stromal keratocytes into their hydrogel.^20^

#### Cell viability and organization

In Mörö et al., cell viability remained > 90% over 28 days, with stable proliferation and preserved keratocyte phenotype.^18^ Fernández-Pérez et al. confirmed keratocyte viability throughout stromal depth prior to implantation, with higher density and retention in long-term pre-cultured grafts.^19^ Vijayaraghavan et al. observed progressive viability increases up to day 14, similar between 0.5% and 1% agarose formulations.^20^

#### Functional assessment and optical properties

Mörö’s bioprinted model achieved >80% light transmittance in the visible range, comparable to human stroma; the construct supported guided axonal growth through the printed channels, forming functional in vitro neural networks extending to the center of the substitute.^18^

In Fernández-Pérez et al., long-term recellularized grafts exhibited greater transparency after implantation in rabbits than short-term or acellular grafts, with better epithelial and stromal integration, and reduced inflammation and neovascularization.^19^

In Vijayaraghavan et al., transparency after 14 days was 75%–80% in the visible range—comparable to human cornea—with stable optical properties over time.^20^

#### Maintenance of cellular phenotype

Mörö’s substitute showed sustained expression of keratocyte markers (keratocan, lumican, ALDH3A1) and neuronal proteins (βIII-tubulin, peripherin).^18^ Fernández-Pérez et al. identified dendritic-morphology keratocytes expressing keratocan without myofibroblast markers.^19^ Vijayaraghavan et al. reported positivity for vimentin and keratan sulfate, confirming phenotype maintenance without fibroblast differentiation.^20^

#### Biomechanical properties

In Mörö et al., elastic modulus and tensile strength were comparable to reported human stromal values and remained stable during culture; compressive modulus (acellular construct) was 10.3 kPa.^18^ Fernández-Pérez et al. observed preserved stiffness after culture and post-implantation, without collagen disorganization.^19^ Vijayaraghavan et al. found predominantly elastic rheology; higher agarose increased stiffness and reduced hydration capacity.^20^

#### In vivo biological response

Only Fernández-Pérez et al. performed in vivo testing: at 3 months, long-term recellularized grafts showed higher transparency, better host integration, and stromal collagen organization closer to physiological, with minimal inflammatory response, some neovascularization, and no intraocular pressure elevation. The construct was easily transplanted in rabbit corneas with standard surgical instruments; anterior-segment OCT confirmed defect repair and proper apposition of substitute to native tissue.^19^

#### Limitations

Despite advances, limitations constrain clinical extrapolation. Mörö’s innervated bioprinted model was evaluated exclusively in vitro, without animal testing or functional validation in human cornea, and lacked surgical handling and long-term degradation assays.^18^ Prolonged-recellularization grafts in Fernández-Pérez used a small sample and short follow-up (three months), insufficient for long-term durability; residual neovascularization persisted, and corneal re-innervation was not evaluated.^19^ Vijayaraghavan’s work was limited to in vitro assays with goat keratocytes; transparency and mechanics were tested under static conditions without long-term degradation; the use of animal cells reduces clinical generalizability.^20^

### Epithelial corneal substitutes

Only one study met the criteria for tissue-engineered epithelial substitutes. Long et al. used AM scaffolds with mesenchymal stem cells (MSCs) and cultured rabbit limbal stem-derived corneal epithelial cells for subsequent transplantation in animal models of limbal deficiency.^21^

After 14 days of culture, a stratified epithelial layer suitable for transplantation developed.^21^ Artificial-tissue cells showed high proliferative and differentiation capacity by immunofluorescence, Western blot, and RT-qPCR.^21^ Transparency was higher in constructs generated with thinner biomaterial; transparency was similar between MSC-containing and MSC-free constructs.^21^

At 14 days post-implantation, only the group receiving an ultrathin AM construct with inoculated MSCs showed a transparent cornea, no inflammatory reaction, a regular epithelium, and negative fluorescein staining, indicating complete re-epithelialization; constructs without MSCs exhibited stromal haze and incomplete epithelialization.^21^

Limitations included short in vivo follow-up (14 days), unspecified animal sample size, interspecies immune differences, and absence of a control group comparable to standard clinical therapy (e.g., limbal graft or AM alone in humans).

### Corneal substitutes including epithelium and stroma

Five articles engineered constructs to replace epithelium and stroma simultaneously, assessing histologic, molecular, functional, and— in some cases—optical and biomechanical parameters. Two papers (Ortiz-Arrabal et al. and González-Gallardo et al.) analyzed the same tissue construct (NANOULCOR).^10,11^

#### Biomaterials and cells used

Ortiz-Arrabal et al. and González-Gallardo et al. characterized NANOULCOR: an ex vivo human cornea generated on nanostructured fibrin-agarose biomaterials seeded with allogeneic human epithelial and stromal cells. González-Gallardo et al. applied the construct in patients with severe trophic corneal ulcers refractory to treatment, some with limbal stem cell deficiency and stromal damage.^10,11^

Bhutani et al. bioprinted biopolymeric corneal lenticules using digital light processing (DLP), encapsulating human corneal stromal cells (hCSCs) to enable integration and remodeling.^22^

Xie et al. created a stromal implant from decellularized allogeneic cornea-derived matrix repopulated with stromal cells for corneal transplantation.^23^

Garzón et al. described two bioengineered corneal models—orthotypic and heterotypic—based on fibrin/agarose biomaterials combined with human corneal cells (orthotypic) or Wharton’s jelly–derived mesenchymal cells (heterotypic), and evaluated long-term in vivo performance.^10,24^

#### Cell viability and organization

All models established an epithelial layer after culture or implantation. In NANOULCOR, constructs developed a stratified five to six-layer epithelium with elongated morphology similar to native cornea, lacking specialized structures such as Vogt’s palisades but forming a well-defined epithelium–stroma interface.^11^ The epithelium remained intact and well-differentiated long-term on the biomaterial surface (transient epithelial defects in three patients were reported by González-Gallardo et al.).^10^

Bhutani et al. showed good viability after encapsulating hCSCs, with no significant cytotoxicity.^22^

Xie et al. reported rapid in vivo epithelial repopulation with complete surface coverage within days.^23^

Garzón et al. observed a mean of ~6.5 epithelial cell layers, with desquamation and active renewal ex vivo and in vivo; no necrosis or loss of integrity occurred over 12 months in rabbits.^24^

#### Functional assessment and optical properties

NANOULCOR reached ~80% transmittance ex vivo in prior group data.^11^ In human in vivo studies, all patients achieved complete and stable surface coverage after surgery; two of five had stable corneal opacity and three of five showed modest visual improvement, though sample size and comorbidities (diabetic retinopathy, glaucoma, retinal detachment) limited statistical significance.^10^ No ulcer recurrence or rescue surgery was required over 24 months.

Bhutani et al. confirmed high transparency across the visible spectrum (optical transmittance 85.45% ± 0.14%), similar to normal native cornea.^22^

Xie et al. reported complete host integration with restored corneal thickness and maintained transparency, reaching 91.46% ± 1.05% transmittance by slit-lamp assessment in their animal model.^23^

Garzón et al. showed a proper epithelial–stromal interface and stable graft–host adhesion. Initial light transmittance was ~60% versus control, increasing over time and higher in the orthotypic model. Expression of crystallins (CRY-αB, CRY-l1, CRY-Z)—key for corneal transparency—increased over time ex vivo, with inter-model differences and partial reduction versus human controls in some groups.^24^

#### Biocompatibility and immunogenicity

In NANOULCOR, human data showed good tolerance in all cases, with no signs of rejection, neovascularization, local infection, or graft detachment; only three transient epithelial defects and one leukoma were reported, none severe.^10^

In animal studies (Garzón and Xie), no immune rejection, tumorigenesis, or infection was observed.^23,24^ Xie et al. noted minimal acute inflammation, good integration, and no significant neovascularization.^23^ Garzón et al. reported mild inflammatory responses during the first 3 months that resolved spontaneously, more marked in the heterotypic model (Wharton’s jelly-derived cells).^24^

#### Stromal organization and differentiation

Histologically, models showed dense, well-organized stroma after prolonged in vivo implantation. In NANOULCOR, fibrin-agarose matrix displayed lower collagen/proteoglycan content and less-ordered architecture than native cornea, yet with in vivo maturation capacity.^11^ In human recipients, anterior-segment OCT showed a more homogeneous surface versus preoperative status; complete scaffold resorption occurred at ~50 days on average, with host tissue replacement.^10^

Bhutani et al. confirmed collagen network preservation compatible with optical/biomechanical function.^22^

Xie et al. showed host keratocyte migration and progressive collagen synthesis, achieving near-native architecture within weeks.^23^

Garzón et al. reported an organized fibrillar mesh with abundant stromal cells at 12 months; the graft–host interface was detectable only during the first 3 months.^24^

#### Maintenance of cellular phenotype

In NANOULCOR, the epithelium strongly expressed cytokeratins (PCK, AE1/AE3, KRT5) with a marker profile (KRT15, KRT19, ΔNp63) closer to the limbal niche than the central cornea, suggesting preserved progenitor phenotype.^11^

Bhutani et al. reported viable encapsulated cells with maintained morphology and extracellular matrix production.^22^

Xie et al. confirmed re-expression of keratocan, lumican, and other stromal proteoglycans by immunohistochemistry.^23^

In Garzón et al., both orthotypic and heterotypic models showed epithelial CK3/12 expression in vivo (even when initially negative in the heterotypic model), strong expression of junctional protein PKG, and stromal marker vimentin; collagen expression increased progressively, peaking at 12 months.^24^

#### Biomechanical properties and surgical handling

After plastic compression nano-structuring, NANOULCOR markedly improved biomechanical properties, facilitating handling and providing sufficient resistance to hold 10-0 nylon sutures without tearing.^10,11^

Bhutani et al. performed puncture resistance tests with 10-0 nylon, showing favorable surgical manipulation; compressive modulus was 535.42 ± 29.05 kPa and hydration/edema was low (2.22 ± 0.36%), favorable for corneal regeneration.^22^

Xie et al. reported adequate flexibility and suture-holding capacity with tensile strength 2.74 ± 0.66 MPa, elongation at break 10.40 ± 1.35%, and Young’s modulus 37.02 ± 2.59 MPa.^23^

Garzón et al. did not report quantitative mechanics but confirmed stability and resistance sufficient to perform DALK.^24^

#### Limitations

Common limitations hinder extrapolation and comparability. Except for NANOULCOR (phase I–IIA human trial), none included large-scale human clinical studies; some relied solely on in vitro culture. Sample sizes were small.

Regarding follow-up, only Garzón and González-Gallardo provide acceptable durations (up to 12 and 24 months, respectively).^10,24^ Others had short follow-up, limiting assessment of long-term behavior and durability.

Only NANOULCOR was compared with a standard technique for persistent, refractory trophic corneal ulcers; the remaining studies did not compare with commercial products or standard care, limiting clinical contextualization.

Despite its promising preclinical and early clinical results, the NANOULCOR model presents several translational limitations. Its manufacture requires specialized GMP-compliant facilities, expanded allogeneic human cells, sequential culture steps, and strict quality-control procedures, which increase logistical complexity and may hinder large-scale implementation. In addition, the product must be transported under controlled conditions and implanted within a limited time window, which may restrict its applicability to highly specialized centers. Although these features support product quality and safety, they may also increase production costs and pose challenges for scalability and inter-center reproducibility. Furthermore, current clinical evidence remains limited and originates largely from a single research program, highlighting the need for independent validation and broader multicenter evaluation before widespread clinical adoption can be considered.

Overall, epithelium–stroma models offer complementary strengths: a construct combining the high structural/biochemical biomimicry of allogeneic matrices, NANOULCOR’s limbal-regenerative capacity, the geometric precision of 3D printing, and the long-term validation of Garzón et al. could closely approximate an optimal, clinically applicable corneal substitute.

A major limitation of this review lies in the substantial heterogeneity among the included studies. The analyzed works differ considerably in terms of animal models (e.g., rabbit, porcine, feline), cell sources (primary human cells, animal-derived cells, mesenchymal stem cells, and induced pluripotent stem cell–derived cells), and biomaterials used for scaffold fabrication.

In addition, there is marked variability in the methods employed to assess optical, functional, and biological outcomes. Parameters such as corneal transparency, thickness, and cellular functionality were measured using different techniques and time points, often without standardized protocols.

This heterogeneity limits the direct comparability between studies and precludes any quantitative synthesis of results. Consequently, the findings of this review should be interpreted with caution, as differences in experimental design may significantly influence reported outcomes.

Future studies would benefit from the adoption of standardized evaluation criteria and reporting guidelines to facilitate cross-study comparison and accelerate clinical translation.

Another important limitation of the present review is the exclusion of acellular stromal laminas, one of the few corneal stromal regeneration strategies translated into clinical practice in humans, as demonstrated by Zarif et al. These studies were not included in the systematic analysis due to the predefined focus of this review on cell-based constructs. This distinction is relevant, as the incorporation of cellular components is considered a key factor for achieving long-term tissue integration, regeneration, and functional restoration. Although this approach allowed a more homogeneous analysis of cell-based strategies, it may have excluded relevant clinical evidence regarding acellular or partially recellularized scaffolds.

## Discussion

This systematic review highlights the significant progress achieved between 2020 and 2025 in the field of corneal tissue engineering, encompassing advances in biomaterials, stem cell technology, and biofabrication methods.

The review was restricted to studies published between January 2020 and June 2025 in order to capture the most recent advances in corneal tissue engineering. This period has been characterized by significant progress in key areas such as 3D bioprinting, stem cell technologies, biomaterial design, and translational approaches toward clinical application. Earlier studies, although foundational, often reflect preliminary stages of development that may not accurately represent the current state-of-the-art or the translational potential of contemporary tissue-engineered corneal substitutes. Therefore, focusing on recent literature allows for a more clinically relevant and up-to-date analysis of emerging strategies with potential applicability in modern ophthalmic practice.

The inclusion of both preclinical and clinical studies was intentional, given the translational nature of corneal tissue engineering. As most advances in this field are still at the experimental stage, limiting the analysis to clinical studies would have excluded a substantial portion of relevant evidence. Therefore, both types of studies were analyzed within a unified framework, focusing on their biological, functional, and translational characteristics rather than direct clinical comparability.

A comparative analysis of multilayered tissue-engineered corneal substitutes shows that different approaches offer complementary strengths and limitations. Biological scaffolds provide high biomimicry but face challenges in standardization and scalability. Decellularized matrices preserve native architecture and optical properties, although variability between samples may affect reproducibility. In contrast, 3D bioprinted constructs offer greater control and reproducibility, but still lack robust in vivo and clinical validation. Overall, no single approach currently fulfills all the requirements for an ideal corneal substitute, highlighting the need for further optimization and comparative studies.

### Endothelial substitutes

Engineered endothelial layers remain among the most extensively studied components.^14–18^ Collagen- and Descemet membrane-based scaffolds support endothelial adhesion, hexagonal morphology, and pump function, with promising results in ex vivo and short-term in vivo models. However, the primary limitations include short follow-up duration, small sample sizes, and limited long-term functionality data.

The differentiation of iPSCs into corneal endothelial-like cells represents an innovative step toward autologous and renewable cell sources, but scalability, purity, and phenotypic stability remain concerns before clinical implementation.^17^

### Stromal substitutes

Stromal constructs, particularly those fabricated by 3D bioprinting, have advanced substantially in mimicking corneal architecture.^19–21^ The incorporation of neuronal elements within the stromal microenvironment marks an important step toward reproducing the corneal sensory network, which is critical for epithelial homeostasis. However, most models were evaluated only under static in vitro conditions. Mechanical, optical, and long-term degradation properties in vivo are still poorly characterized.

Decellularized xenogeneic matrices have shown excellent transparency and low immunogenicity, yet standardization of decellularization protocols and sterility control are required for regulatory acceptance.

### Epithelial substitutes

The epithelial surface is essential for maintaining transparency and protection. The single epithelial study identified in this review showed that MSCs enhance epithelialization and reduce inflammation in a rabbit model of limbal deficiency.^10^ Despite these findings, the evidence is still preliminary, and human clinical data are lacking.

Future research should aim to integrate epithelial stem cells derived from limbal niches or induced pluripotent sources onto clinically approved scaffolds with sufficient transparency and handling properties.

### Epithelium–stroma composite constructs

Multilayered constructs represent the most comprehensive and translationally relevant approach. The NANOULCOR model and related fibrin–agarose scaffolds have demonstrated long-term biocompatibility and integration both in preclinical and early human trials.^10,11,24^

These models combine the biomimetic structure of biological hydrogels with clinical feasibility, achieving suturability, progressive transparency, and re-epithelialization. Nevertheless, the small number of treated patients and the heterogeneity of underlying pathologies limit statistical power.

The relative prominence of the NANOULCOR model within this review is primarily related to the availability of more advanced translational data compared to other approaches, including preclinical validation and early clinical application. Nevertheless, it is important to emphasize that the current evidence remains limited in scale and originates predominantly from a single research group. Therefore, caution is warranted when interpreting these findings, and further independent validation studies are required to confirm reproducibility, scalability, and long-term clinical outcomes.

Other 3D-printed or decellularized constructs showed comparable biomechanical properties to native cornea and may complement biological scaffolds with greater reproducibility.^22,23^

### Translational barriers and future perspectives

Despite considerable advances, several barriers still hinder clinical translation:

- The majority of studies rely on animal models, which cannot fully replicate human corneal physiology.
- Follow-up durations are generally short (<3 months).
- There is a lack of comparative studies against standard keratoplasty or limbal grafts.
- Regulatory pathways for advanced therapy medicinal products (ATMPs) remain complex, particularly for xenogeneic or composite biomaterials.

Emerging trends such as bio-printed lamellar scaffolds, smart hydrogels with controlled degradation, and CRISPR-based cell optimization may accelerate progress toward fully functional, patient-specific corneal equivalents.

Regulatory considerations represent a major challenge for the clinical implementation of tissue-engineered corneal substitutes. Constructs combining living cells with biomaterial scaffolds are typically classified as advanced therapy medicinal products (ATMPs) under regulatory frameworks such as those of the European Medicines Agency (EMA). This classification entails stringent requirements regarding manufacturing, quality control, safety, and clinical validation.

In particular, production must be carried out in Good Manufacturing Practice (GMP)-compliant facilities, ensuring sterility, batch consistency, and traceability of biological materials. Additionally, variability in cell sources, biomaterials, and fabrication processes complicates standardization and reproducibility across centers. These requirements, together with high production costs and logistical constraints, may limit the widespread clinical adoption of tissue-engineered corneal substitutes despite their promising therapeutic potential.

Addressing these challenges will be essential for the successful clinical translation of tissue-engineered corneal substitutes.

An important consideration when interpreting the findings of this review is the existence of clinically validated corneal stromal regeneration approaches based on acellular or partially recellularized stromal laminas, particularly those developed by El Zarif et al. These studies represent some of the few examples of successful clinical translation in humans. In particular, El Zarif et al. reported long-term outcomes following intrastromal implantation of autologous adipose-derived adult stem cells (ADASCs), decellularized stromal laminas, and ADASC-recellularized laminas in patients with advanced keratoconus, demonstrating moderate visual improvement and a favorable safety profile at 3 years.^25^ Notably, even in the absence of exogenous cells, decellularized laminas showed progressive in vivo repopulation by host keratocytes, suggesting an intrinsic regenerative capacity of the corneal stroma. This phenomenon has also been supported by confocal microscopy analyses showing increased stromal cellularity and morphological adaptation toward keratocyte-like phenotypes over time. Furthermore, objective evaluation of corneal transparency has also been reported in this regenerative model. Using corneal densitometry analysis, El Zarif et al. demonstrated a progressive reduction in stromal light scatter and improvement in corneal transparency following implantation of human acellular stromal laminas, supporting the concept that optical restoration may continue beyond the initial postoperative period as stromal remodeling progresses. These findings provide quantitative evidence of functional tissue integration and represent one of the few objective assessments of transparency outcomes currently available in the field of corneal bioengineering.^26^

These approaches were not included in the systematic analysis of this review due to the predefined focus on tissue-engineered constructs incorporating living cells at the time of implantation. This methodological decision was intended to allow a more homogeneous evaluation of cell-based bioengineered corneal substitutes.

However, the exclusion of these clinically validated techniques represents a limitation, as they provide important evidence regarding stromal regeneration and highlight the translational gap between experimental tissue-engineered constructs and approaches already implemented in clinical practice.

Notably, the ability of acellular laminas to undergo in vivo repopulation by host keratocytes suggests that effective corneal regeneration may also be achieved through host-driven mechanisms, without the need for exogenous cellular components. This contrasts with cell-based tissue-engineered approaches, which aim to enhance regeneration through active biological participation of implanted cells.

Therefore, both strategies should be considered complementary rather than mutually exclusive, and future research should aim to integrate their respective advantages to optimize clinical outcomes.

## Conclusion

Recent years have witnessed remarkable progress in the development of tissue-engineered corneal substitutes, offering viable alternatives to traditional donor transplantation and keratoprosthesis. The integration of advanced biomaterials, stem-cell-based approaches, and biofabrication technologies—such as 3D bioprinting and nanostructured fibrin-agarose scaffolds—has enabled the creation of constructs that closely mimic the native corneal microarchitecture and function.

However, despite promising preclinical and early clinical results, several challenges remain before these bioengineered substitutes can be routinely implemented in ophthalmic practice. Most studies are still limited by short follow-up periods, small sample sizes, and reliance on animal models. Furthermore, the long-term biomechanical stability, transparency, and immunological behavior of these constructs in humans require deeper investigation.

Future efforts should focus on standardizing fabrication protocols, conducting large-scale clinical trials, and ensuring regulatory compliance for advanced therapy medicinal products (ATMPs). Interdisciplinary collaboration among ophthalmologists, biomaterials scientists, and tissue engineers will be crucial to translate these laboratory breakthroughs into safe and effective therapies.

In summary, tissue-engineered corneal models represent a rapidly evolving and highly promising frontier in regenerative ophthalmology, bringing us closer to a sustainable and immunocompatible solution for global corneal blindness.

## Supplemental Material

sj-docx-1-oed-10.1177_25158414261464536 – Supplemental material for State-of-the-art advances in tissue-engineered corneal substitutes: a systematic review (2020–2025) Supplemental material, sj-docx-1-oed-10.1177_25158414261464536 for State-of-the-art advances in tissue-engineered corneal substitutes: a systematic review (2020–2025) by Mario Bonmatí-Echevarría, Carmen González-Gallardo and Miguel Alaminos in Therapeutic Advances in Ophthalmology

## Declarations

**Ethics approval and consent to participate:** Not applicable.

**Consent for publication:** Not applicable.

**Availability of data and materials:** The datasets generated and analyzed during the current study are not publicly available due to the nature of this systematic review, which is based on previously published studies. No analytic code was used. Data extraction forms and extracted data are available from the corresponding author upon reasonable request.*
